# Squamous cell carcinoma mimics small cell carcinoma of the lung: a case report

**DOI:** 10.1093/jscr/rjaa531

**Published:** 2020-12-28

**Authors:** Michael Kmeid, Breanne Gillie, Armand Asarian, Philip Xiao

**Affiliations:** St George’s University School of Medicine, True Blue, Grenada; St George’s University School of Medicine, True Blue, Grenada; Department of Surgery, The Brooklyn Hospital Center, Icahn School of Medicine at Mount Sinai, Brooklyn, NY 11201, USA; Department of Pathology, The Brooklyn Hospital Center, Icahn School of Medicine at Mount Sinai, Brooklyn, NY 11201, USA

## Abstract

Squamous cell carcinomas (SCC) accounts for roughly 20% of lung cancers in the USA. The 2015 World Health Organization classification of lung tumors further categorizes SCC as three subtypes: keratinizing, non-keratinizing and basaloid variant. The non-keratinizing subtype is a poorly differentiated tumor that can present histologically in different ways, and one of which is a rare variant that strongly resembles small cell carcinoma. As a result, histological diagnosis alone is not sufficient to properly diagnose lung carcinomas. Immunohistochemistry has been increasingly used over the past few years to differentiate between lung tumors. The combination of morphological and immunohistochemical staining should be the mainstay for diagnosis of all lung carcinomas as more targeted therapies become more available.

## INTRODUCTION

Squamous cell carcinomas (SCC) make up ~20% of all lung cancers diagnosed in the USA every year [1].The 2004 World Health Organization (WHO) classification recognized four variants of the lung squamous cell carcinoma: clear cell, small cell, papillary and basaloid [2]. However, because the small cell and clear cell variants were rare, these terms were changed in 2015, and SCCs were reclassified as basaloid and keratinizing or non-keratinizing, based on the degree of histologic differentiation, well or poorly differentiated, respectively [3, 4]. Historically, the gold standard for diagnosing the lung carcinoma subtypes was via the morphological differences seen on light microscopy using hematoxylin and eosin (HE) stain [5]. SCC is identified on light microscopy by the presence of keratinization and intercellular bridges [5]. However, this method is complicated by the poorly differentiated non-keratinizing carcinomas, which do not include this characteristic histologic appearance and make it challenging to differentiate from the histology of primary small cell carcinoma of the lung [3]. Thus, lung tumor diagnosis requires the addition of immunohistochemistry, which plays an essential role in differentiating biopsy specimens that share the same histologic characteristics [3]. The aims of this case report are to (i) report and review a case of a patient diagnosed with SCC poorly differentiated non-keratinizing subtype; (ii) evaluate histological morphology and immunohistochemistry of SCC poorly differentiated non-keratinizing subtype and (iii) comment on the importance of immunohistochemical (IHC) confirmation in the diagnosis of lung tumors.

## CASE REPORT

**Figure 1:**
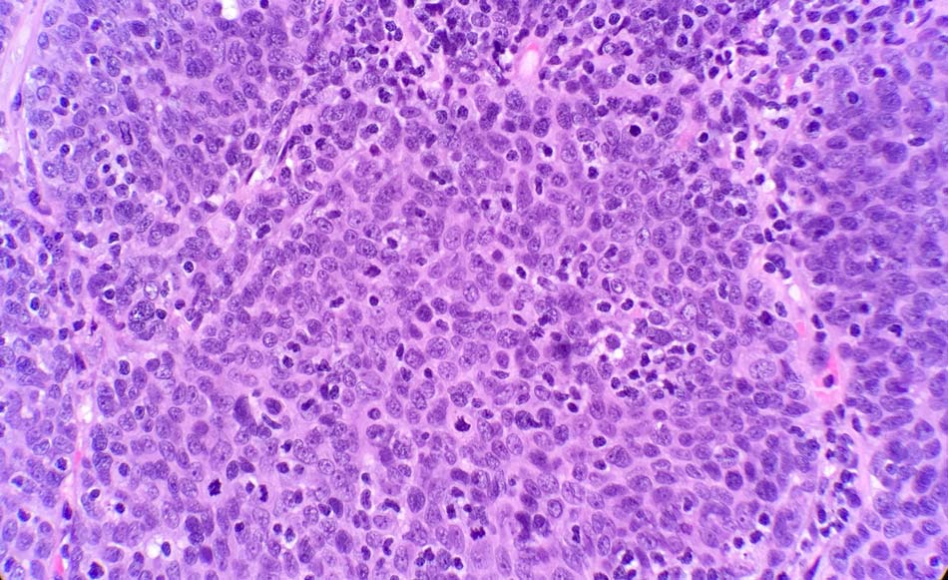
Microscopic examination reveals nest of small blue cells with high N/C ratio (HE ×40).

**Figure 2:**
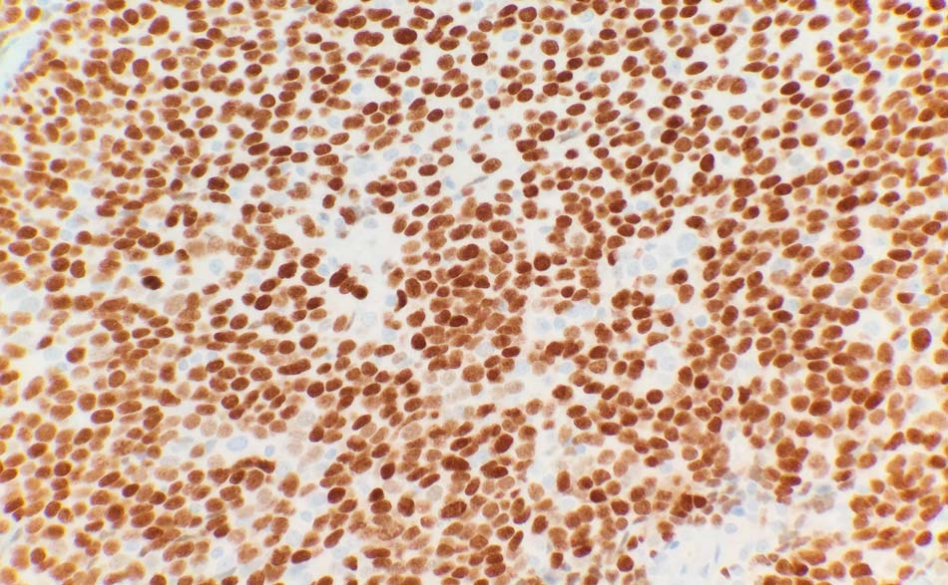
Tumor cells are immunopositive for p63 (IHC ×40).

**Figure 3:**
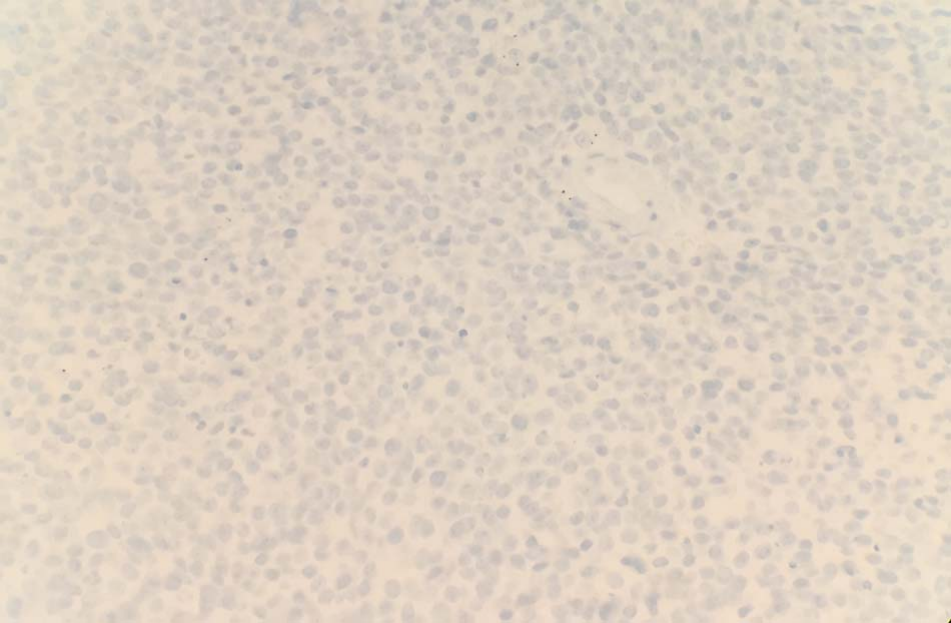
Tumor cells are immunonegative for chromogranin (IHC ×40).

The patient had a past medical history of hypertension, diabetes mellitus type 2 and hyperlipidemia. Three years before this, the patient was diagnosed with left lung small cell carcinoma on cytology and underwent chemotherapy with surveillance screening. The patient presented for a left lung biopsy and left subclavian mediport placement following the detection of a lingular nodule. Grossly, the lingular nodule specimen measured 3.3 × 3.0 × 1.4 cm, and cut sections showed a tan mass measuring 1.5 × 2 cm. Microscopic examination revealed that the tumor was comprised of nests of small blue cells with high nuclear/cytoplasmic (N/C) ratio and high mitotic activity (Fig. 1). No morphological features of squamous differentiation were seen. Tumor cells have distinct nucleoli and no molding make small cell carcinoma less likely. IHC stains reveal that tumor cells were positive for squamous cell markers (p40, p63 and CK5/6) (Fig. 2), Pan Keratin, TTF1 (focal) and negative for neuroendocrine markers (CD56, chromogranin and synaptophysin) (Fig. 3) and CD45. Ki 67 immunostain has ~70% positivity. Combined with morphological features, this immunochemical profile supported a diagnosis of the poorly differentiated non-keratinizing squamous cell carcinoma rather than small cell carcinoma.

## DISCUSSION

Histologic identification with HE stain is a reliable method for diagnosing well-differentiated keratinizing-type SCC tumors with clear morphological patterns, including keratinization and intercellular bridges [3]. However, it is not satisfactory for distinguishing between poorly differentiated histologic subtypes of non-small cell carcinoma, and additional IHC stain is recommended for diagnosis [3]. In comparison, the current WHO recommendation for the diagnosis of small cell carcinoma is morphology alone, with immunohistochemistry indicated as a useful adjuvant but not mandatory for diagnosis [6]. Some limitations of small cell carcinoma morphological diagnosis include crush artifacts, tumor necrosis, limited tumor presentation and difficulty distinguishing between small cell carcinoma and the poorly differentiated non-keratinizing variant of squamous cell carcinoma, as seen in the case of our patient [7]. With this in mind, immunohistochemistry may be a critical adjuvant in the diagnosis of squamous cell carcinoma.

Most small cell carcinoma display a TTF-1+/p63−/high molecular weight keratin− immunophenotype, whereas the poorly differentiated non-keratinizing subtype of SCC shows opposite reactivity displaying a TTF-1/p63+/high molecular weight keratin+ immunophenotype [7]. In particular, p63 staining is inversely related to keratinization, making p63 an important marker for squamous differentiation in the poorly differentiated squamous cell carcinoma [7].

As is evidenced in this case study, the use of morphologic diagnosis alone may lead to improper diagnosis as it poorly differentiates between small cell carcinoma and the poorly differentiated non-keratinizing subtype of squamous cell carcinoma. Distinguishing between these tumors becomes significantly more important as advances in chemotherapy are made, and targeted immune therapies become more widely available [3]. Targeted immunotherapeutic agents for SCC approved for use by the Food and Drug Administration include the PD1 antagonist pembrolizumab, and research for other targetable pathways and receptors, including FGFR and the PI3K–AKT pathway, is ongoing [8]. In contrast, anti-apoptotic Bcl-2 proteins and self-renewal pathways such as hedgehog and NOTCH have been identified as potential targets for small cell carcinoma treatment [9].

## CONCLUSION

The poorly differentiated non-keratinizing variant of SCC is an unusual subtype of SCC. It strongly resembles small cell carcinoma histologically and can be easily misdiagnosed without the use of immunohistochemistry. A panel of TTF-1, p63 and high molecular weight keratin is a useful supplement to histological examination for distinguishing between the two tumors. The poorly differentiated non-keratinizing variant of SCC is TTF-1−/p63+/high molecular weight keratin+/differentiating it from small cell carcinoma, which is TTF 1+/p63−/high molecular weight keratin−. A combination of histology and IHC staining should be routinely used in clinical practice to ensure proper differentiation between the subtypes. As more targeted immune therapies for lung cancer treatment become available, distinguishing these subtypes will be critical in their management.

## CONFLICT OF INTEREST STATEMENT

None declared.
